# Liver Cancer in Guatemala: An Analysis of Mortality and Incidence Trends From 2012 to 2016

**DOI:** 10.1200/JGO.18.00179

**Published:** 2019-03-05

**Authors:** Alba J. Kihn-Alarcón, María F. Toledo-Ponce, Angel Velarde, Ximing Xu

**Affiliations:** ^1^ Instituto Nacional de Cancerología, Guatemala City, Guatemala; ^2^ Renmin Hospital of Wuhan University, Wuhan, People’s Republic of China; ^3^ Zhongnan Hospital of Wuhan University, Wuhan, People’s Republic of China

## Abstract

**PURPOSE:**

Guatemala has the highest mortality and incidence of liver cancer in Central and South America. The aim of this study is to describe the extent of liver cancer in the country from 2012 to 2016 and the associated risk factors.

**METHODS:**

A secondary analysis was performed using liver cancer mortality and morbidity data and data on risk factors, such as hepatitis B virus infection, cirrhosis, and alcoholism.

**RESULTS:**

Analysis revealed that liver cancer causes approximately 20% of cancer deaths in the country, is more frequent in the population older than age 65 years old, and is increasing in those age 30 to 44 years. More than 25% of deaths occurred in the North and West regions. The incidence of major risk factors for development of liver cancer has decreased.

**CONCLUSION:**

The high mortality of liver cancer compared with its incidence indicates that most patients are diagnosed at late stages. To reduce the burden of liver cancer, creation of strategies for earlier detection is needed.

## INTRODUCTION

Worldwide, every year, there are nearly 800,000 patients diagnosed with liver cancer; it is the sixth most common malignancy and the third most lethal malignancy.^1,2^ Almost three quarters of new patients live in areas with low- and medium-income areas.^1^ Liver cancer can occur in association with chronic hepatitis B virus (HBV) infection in the presence or absence of cirrhosis.^3^ Other risk factors include hepatic cirrhosis of any etiology, excessive consumption of alcohol, exposure to aflatoxins, certain inherited diseases, and metabolic disorders such as obesity, diabetes, and nonalcoholic fatty liver disease.^4^

Guatemala is a country of more than 16 million inhabitants, according to the Guatemalan National Institute of Statistics, and has a low- to middle-income economy. In Guatemala, noncommunicable diseases account for approximately half of total deaths, with cancer accounting for approximately 12% of all deaths.^5^ Guatemala has the highest incidence and mortality rates of all types of malignancies in Central and South America, including liver cancer, with a unique 1:1 distribution in men and women.^6-8^

Compared with other areas of the world where the highest incidence rates of liver cancer are in regions with the highest prevalence of predisposing conditions such as chronic HBV and hepatitis C virus (HCV) infections, according to previous studies, Guatemala has a low endemicity (less than 2%) of carriers of HBV.^1,9,10^ It is also estimated that there are approximately 145,000 persons infected with HCV, but approximately 80% are unaware that they carry the virus.^11,12^

Since 2013, the Health Ministry has required that general practitioners and specialists report new cancer cases every 3 months.^13^ In addition, the National Commission for the Prevention of Noncommunicable Diseases and Cancer was created by the Health Ministry with the aim to develop policies and actions to decrease the incidence, prevalence, mortality, and disability associated with noncommunicable diseases, counting on technical and financial support from the Pan American Organization of Health and the Institute of Nutrition of Central America and Panama. The program has developed a guideline for the prevention and management of cancer in the first and second levels of attention in the country; in this guideline, it is mentioned that α-fetoprotein and ultrasound should be used for the diagnosis of liver cancer. Guatemala lacks in most areas the resources for these diagnostic tests; thus, screening the populations at risk for developing liver cancer is not possible.^14^

Despite the high mortality rate in Guatemala as a result of liver cancer, little is known about the risk factors in this population or regional distribution of the disease, which makes it difficult to create a program for prevention and screening for people at risk. In this context, we analyzed the national data available, not just regarding liver cancer, but also regarding the risk factors related to liver cancer, such as alcoholism, cirrhosis, and HBV infection. We also discuss the HBV immunization program.

## METHODS

We performed a secondary analysis from 2012 to 2016 using official national sources of information. For both mortality and morbidity of liver cancer, we included the International Classification of Diseases, 10th Revision, codes for liver cancer (C22, C22.1 to C22.9) and liver carcinoma in situ (D01.5). HBV, cirrhosis, and alcoholism data were obtained from the Work Memory from 2012 to 2015, along with data for immunizations; 2016 data were unavailable at the time of this study. All the data were obtained from the Guatemalan Health Ministry, available online in the Health Information System.^15^ For the calculations of mortality and incidence, we used population data from the National Institute of Statistics. For age-specific mortality and incidence rates, we divided the populations in quinquennial age groups. All graphs and tables were generated using spreadsheets in Microsoft Office Excel 2013 (Microsoft, Redmond, WA), and a map of the country was created using Microsoft Paint on the basis of percentages of deaths as a result of liver cancer per department in the year 2016.

## RESULTS

### Mortality of Liver Cancer

**FIG 1:**
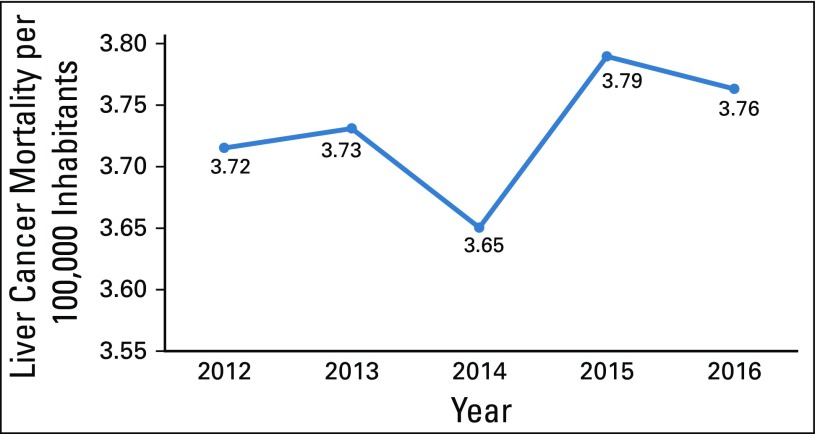
Liver cancer mortality (crude rate) per 100,000 inhabitants in Guatemala from 2012 to 2016.

In Guatemala, cancer is a leading cause of death, accounting for 7.46% of all deaths in 2016. Over a period of 5 years, there was a decrease in cancer-related deaths from 23 to 21 per 100,000 inhabitants. Although deaths as a result of all cancers decreased in the general population, the liver cancer mortality rate showed a tendency to increase and accounted for approximately 20% of all deaths related to malignant tumors (Table 1 and Fig 1).

**TABLE 1:**
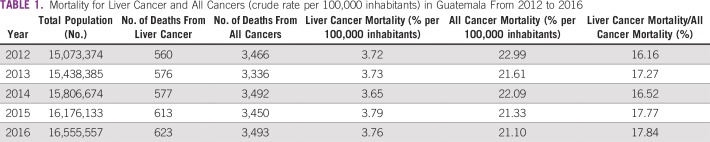
Mortality for Liver Cancer and All Cancers (crude rate per 100,000 inhabitants) in Guatemala From 2012 to 2016

**FIG 2:**
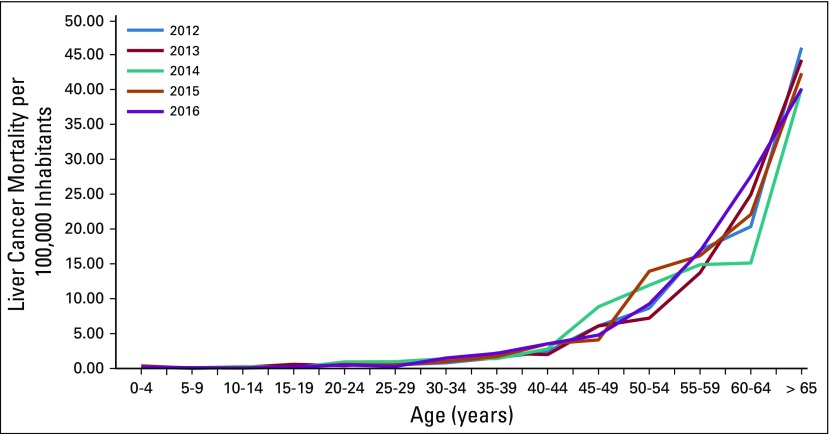
Age-specific liver cancer mortality (crude rate) per 100,000 inhabitants in Guatemala from 2012 to 2016.

**FIG 3:**
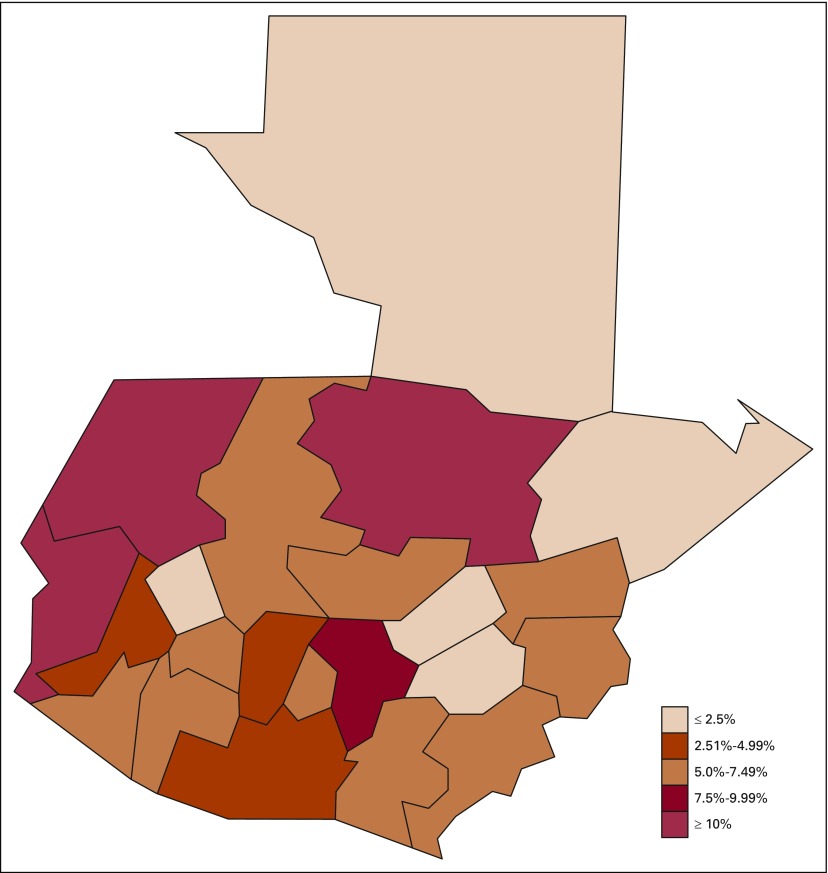
Distribution of liver cancer deaths in Guatemala in 2016.

On average, liver cancer rates have a female-to-male ratio of 1:1 for all age groups, and the age group with the highest percentage of reported deaths (41.24%) was the population older than age 65 years (Table 2 and Fig 2). Approximately three quarters of deaths were reported in people older than age 54 years; however, although liver cancer rates seem to be decreasing from previous years for this population, an increase of 50% in liver cancer–related death was seen in the population between 30 and 44 years old in 2016 compared with 2012 (Table 2 and Fig 2). Most liver cancer–related deaths have occurred in the departments of San Marcos, Alta Verapaz, and Huehuetenango, contributing to more than 30% of all deaths as a result of this malignancy in 2016 (Table 3 and Fig 3).

**TABLE 2:**
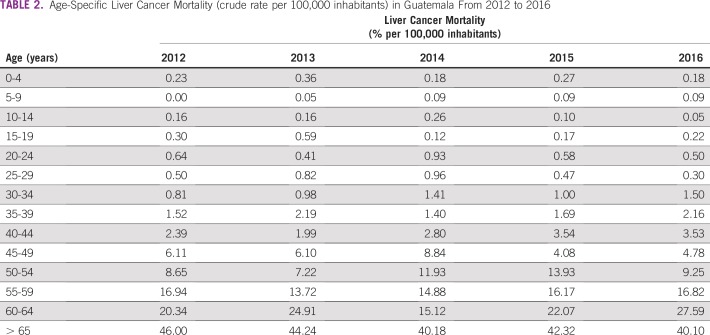
Age-Specific Liver Cancer Mortality (crude rate per 100,000 inhabitants) in Guatemala From 2012 to 2016

**TABLE 3:**
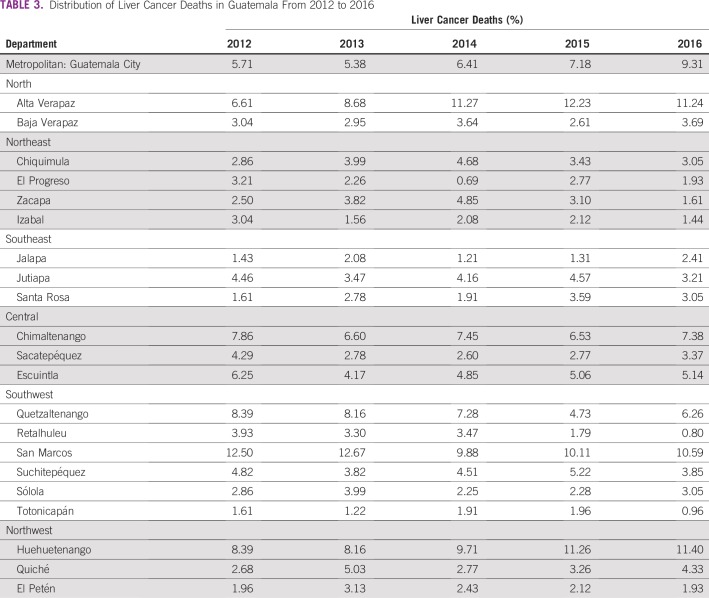
Distribution of Liver Cancer Deaths in Guatemala From 2012 to 2016

### Incidence of Liver Cancer

**FIG 4:**
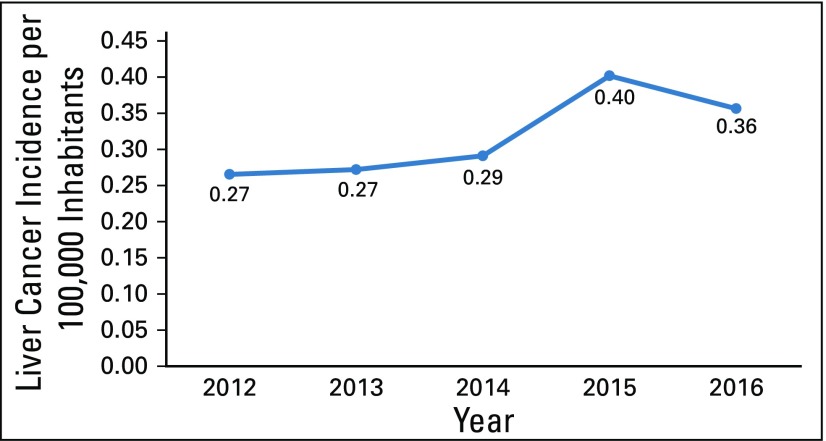
Liver cancer incidence rates per 100,000 inhabitants in Guatemala from 2012 to 2016.

**FIG 5:**
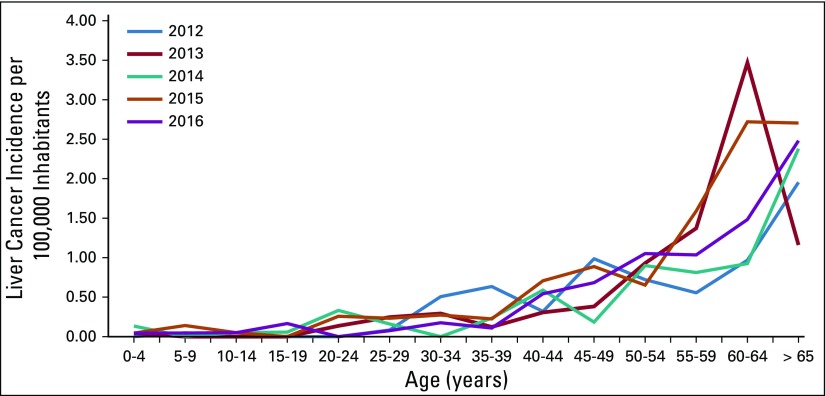
Age-specific liver cancer incidence rates per 100,000 inhabitants in Guatemala from 2012 to 2016.

Unlike for cervical cancer, liver malignancies lack a strategy for early detection and screening of the at-risk population; however, we still analyzed the available information. The incidence of cancer has remained steady among the population, with 11 cancers per 100,000 inhabitants (Table 4 and Fig 4). The incidence of liver cancer has increased approximately 33% from 2012, and liver cancer only represents approximately 3% of all malignancies detected, with six of 10 patients being age 55 years or older (Table 5 and Fig 5).

**TABLE 4:**
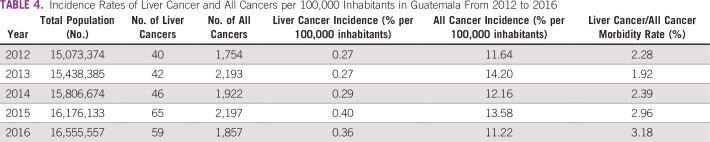
Incidence Rates of Liver Cancer and All Cancers per 100,000 Inhabitants in Guatemala From 2012 to 2016

**TABLE 5:**
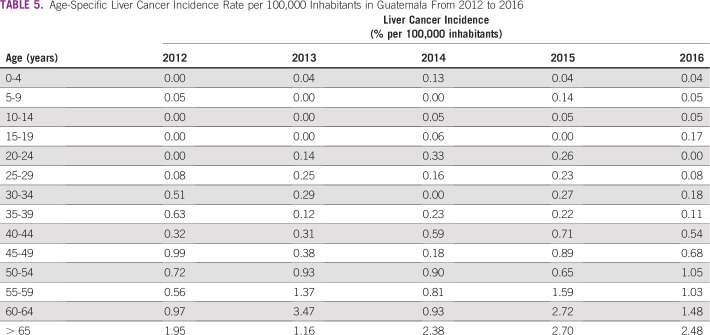
Age-Specific Liver Cancer Incidence Rate per 100,000 Inhabitants in Guatemala From 2012 to 2016

### Incidence and Mortality of Risk Factors for Liver Cancer

**FIG 6:**
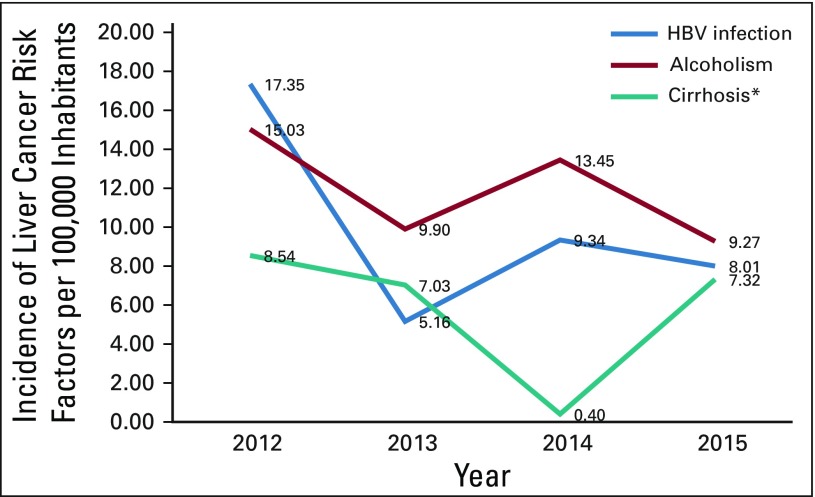
Incidence (per 100,000 inhabitants) of risk factors for liver cancer in Guatemala from 2012 to 2015. HBV, hepatitis B virus. *Cirrhosis mortality rate.

In Guatemala, since 2012, there has been a decrease in the incidence of major risk factors for the development of liver cancer (Table 6 and Fig 6). Currently, in contrast with HCV, there is epidemiologic surveillance of HBV infections, and the incidence of HBV has been reduced by half in Guatemala (Table 6 and Fig 6).

**TABLE 6:**
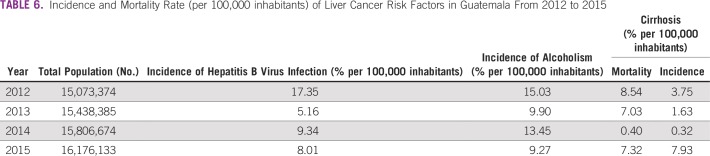
Incidence and Mortality Rate (per 100,000 inhabitants) of Liver Cancer Risk Factors in Guatemala From 2012 to 2015

Since 2013, the National Health System has offered free immunization against HBV along with bacillus Calmette-Guérin at birth, with a coverage rate of 29% among new births. In contrast, the pentavalent vaccine covers more than three quarters of children younger than 1 year old with a complete schedule, but less than 5% of children have received a booster dose by the age of 1 to 6 years .

Liver cirrhosis incidence and mortality rates experienced a dramatic decrease in 2014, but its incidence has increased more than 200% over a period of 4 years (2012 to 2016). In contrast, alcoholism has shown a reduction in incidence among the Guatemalan population (Fig 6).

## DISCUSSION

By 2030, Guatemala will have more than 20 million inhabitants, and as the population ages and the nativity rates decline in Central America, it is expected that the incidence of all cancers will increase by approximately 73%.^4^ The data presented here demonstrate that despite a reduction in cancer-related deaths among the population, the incidence is steady, which will lead to an overwhelming burden for the health system. In addition, although the incidence of liver cancer seems low compared with that of other malignancies, its high mortality indicates that few patients are diagnosed early in the course of the disease. The reasons for this could include the lack of access to health services, low knowledge in the population about the disease, and perceptions about the diagnosis of cancer.

According to 2015 data from The World Bank, 59.1% of Guatemalans live in poverty. The diagnosis of a malignancy could lead to additional impoverishment as a result of its effects on the daily economic situation, often related to the loss of income and health care expenses. To reduce the burden of this malignancy in Guatemala, it is important to create a screening program for the population at risk, including patients with cirrhosis, alcoholism, and HBV or HCV. It is also crucial to investigate whether there are other risk factors associated with liver cancer in Guatemalans, such as aflatoxin exposure and genetic variations. This might help us understand why the disease is increasing among young adults and would provide valuable information that could allow for the creation of measures to prevent the disease or identify patients at an earlier stage.

However, an excellent screening program is only appropriate if there are effective treatment measures that are accessible to patients. Although chemotherapy and radiotherapy are available to the public, only four public or government-supported hospitals offer these treatments in Guatemala, and all of them are located in Guatemala City. Approximately 80% of the population lives outside of the capital of Guatemala City; thus, for many patients, treatment is out of reach or requires traveling long distances.^16-18^ Considering the fact that most patients are diagnosed with late-stage disease, many patients need palliative care, but currently, there are no policies that guarantee access to palliative care to patients, causing unnecessary pain for the patients and their families.^19-21^

Liver cancer incidence is low compared with that of other malignancies in the country, but its high mortality rate indicates that few patients are diagnosed before developing late-stage disease. Reducing the burden of liver cancer in Guatemala requires not only making screening more available for the population at risk, but also identifying other risk factors to enact prevention measures to decrease the risk of the disease.

## References

[1] Ervik M Lam F Ferlay J et al Cancer Today Lyon, France International Agency for Research on Cancer 2016

[2] Smith JW Kroker-Lobos MF Lazo M et al Aflatoxin and viral hepatitis exposures in Guatemala: Molecular biomarkers reveal a unique profile of risk factors in a region of high liver cancer incidence PLoS One 12 e0189255 2017 29236788 10.1371/journal.pone.0189255 PMC5728519

[3] El-Serag HB Epidemiology of viral hepatitis and hepatocellular carcinoma Gastroenterology 142 1264 1273.e1 2012 22537432 10.1053/j.gastro.2011.12.061 PMC3338949

[4] Piñeros M Frech S Frazier L et al Advancing reliable data for cancer control in the Central America Four region J Glob Oncol 2018doi:10.1200/JGO.2016.008227 10.1200/JGO.2016.008227 PMC6180802 30241165

[5] World Health Organization: Noncommunicable diseases country profiles: Guatemala 2014. https://www.who.int/nmh/countries/2018/gtm_en.pdf?ua=1

[6] Wong MC Jiang JY Goggins WB et al International incidence and mortality trends of liver cancer: A global profile Sci Rep 7 45846 2017 28361988 10.1038/srep45846 PMC5374459

[7] Sierra MS Soerjomataram I Antoni S et al Cancer patterns and trends in Central and South America Cancer Epidemiol 44 S23 S42 2016 suppl 1 27678320 10.1016/j.canep.2016.07.013

[8] Bray F Piñeros M Cancer patterns, trends and projections in Latin America and the Caribbean: A global context Salud Publica Mex 58 104 117 2016 27557369 10.21149/spm.v58i2.7779

[9] Tanaka J Hepatitis B epidemiology in Latin America Vaccine 18 S17 S19 2000 suppl 1 10683537 10.1016/s0264-410x(99)00455-7

[10] Akinyemiju T Abera S Ahmed M et al The burden of primary liver cancer and underlying etiologies from 1990 to 2015 at the global, regional, and national level: Results from the Global Burden of Disease Study 2015 JAMA Oncol 3 1683 1691 2017 28983565 10.1001/jamaoncol.2017.3055 PMC5824275

[11] Samayoa B Anderson MR Alonso Pacheco KP et al Seroprevalence of HIV, hepatitis B, and syphilis among pregnant women at the general hospital, Guatemala City, 2005-2009 J Int Assoc Physicians AIDS Care (Chic) 9 313 317 2010 20841440 10.1177/1545109710376669

[12] Pineda Grajeda N Navas S Meléndez J et al Caracterización de factores asociados a la infección por el virus de la hepatitis C Guatemala 2017 [in Spanish] Revista de Medicina Interna de Guatemala 21 7 13 2017

[13] Gobern L, Palacios Cacacho E, Mayen M, et al: Análisis de situación epidemiológica de las enfermedades no transmisibles Guatemala 2015. http://epidemiologia.mspas.gob.gt/files/Publicaciones%202016/Salas%20Situacionales/An%C3%A1lisis%20de%20Enfermedades%20No%20Transmisibles%202015.pdf

[14] National Health Ministry Guatemala: Guía de prevención y atención integral de cáncer. https://www.iccp-portal.org/system/files/plans/GTM_B5_GuiaCancer2016.pdf

[15] National Health System Health Information System 2012. https://sigsa.mspas.gob.gt/

[16] National Health Ministry Guatemala: Vigilancia epidemiológica del cáncer. http://epidemiologia.mspas.gob.gt/files/Publicaciones%202016/Protocolos/Protocolo%20de%20Cancer%20%20junio%202016.pdf

[17] Pan American Health Organization: Cancer in the Americas, country profiles 2013: Guatemala. https://www.paho.org/hq/dmdocuments/2014/GUATEMALA-CANCER-PROFILE-2013.pdf

[18] Human Rights Watch: “Punishing the patient”: Ensuring access to pain treatment in guatemala. https://www.hrw.org/report/2017/05/17/punishing-patient/ensuring-access-pain-treatment-guatemala

[20] Duarte Juárez ER Samayoa Morales VR Liere de Goday AM Propuesta de una Política Nacional de Cuidados Paliativos para Pacientes con Cáncer Guatemala City, Guatemala Universidad de San Carlos de Guatemala, National Institute of Chemical and Biological Research 2013

[21] Human Rights Watch: World report 2013: Events of 2012. https://www.hrw.org/report/2013/01/31/world-report-2013/events-2012

